# Low Blood Glucose and Its Relation to Electrolyte Derangements and Metabolic Acidosis in Critically Ill Children in Malawi

**DOI:** 10.4269/ajtmh.25-0635

**Published:** 2026-03-31

**Authors:** Ance Kreslins, Fatsani Ngwalangwa, Josephine Langton, Ethwako Mlia Phiri, Carina King, Helena Hildenwall

**Affiliations:** ^1^ Department of Global Public Health, Karolinska Institutet, Stockholm, Sweden;; ^2^ Astrid Lindgren Children’s Hospital, Karolinska University Hospital, Stockholm, Sweden;; ^3^ Department of Epidemiology and Biostatistics, Kamuzu University of Health Sciences, Blantyre, Malawi;; ^4^ Department of Pediatrics and Child Health, Queen Elizabeth Central Hospital and Kamuzu University of Health Sciences, Blantyre, Malawi;; ^5^ Department of Clinical Science, Intervention and Technology, Karolinska Institutet, Stockholm, Sweden

## Abstract

Critically ill children with low blood glucose concentrations in low-income settings suffer a high risk of mortality independent of other signs of disease severity and despite administration of the currently recommended treatment of hypoglycemia. This study explores other pathways that may relate to the high mortality risk, namely electrolyte derangements, metabolic acidosis, and hyperketonemia. Children ages 1 month to 5 years old presenting with one or more WHO emergency signs were recruited upon arrival to the Pediatric Accident and Emergency Department at Queen Elizabeth Central Hospital in Malawi between November 2022 and June 2024. A total of 12 hypoglycemic (blood glucose <2.5 mmol/L), 36 low glycemic (blood glucose 2.5–4.9 mmol/L), and 201 normoglycemic (blood glucose 5.0–10.0 mmol/L) children were enrolled. Low glycemic children had increased odds of acidosis (adjusted odds ratio [aOR] = 5.84, 95% CI: 1.17–29.2 for moderate acidosis; aOR = 27.1, 95% CI: 6.08–120.9 for severe acidosis). Among children with acidosis, there were increased odds of hypokalemia (aOR = 24.5, 95% CI: 2.91–205.88), hyponatremia (aOR = 2.58, 95% CI: 1.33–4.99 for moderate acidosis; aOR = 3.62, 95% CI: 1.71–7.68 for severe acidosis), and hypernatremia (aOR = 9.42, 95% CI: 2.50–35.5 for severe acidosis). There were no increased odds of potassium or sodium derangements in those with low glucose concentrations. Low blood glucose concentrations were associated with metabolic acidosis, which in turn, was associated with hypokalemia and hyponatremia/hypernatremia. However, there was no direct association between low blood glucose and electrolyte derangements.

## INTRODUCTION

Hypoglycemia, defined by the WHO as blood glucose concentration <2.5 mmol/L in a well-nourished child (alterative definition: <2.2 mmol/L, which is mainly used in severe malaria),^1^^,^^2^ is a medical emergency affecting approximately 3–7% of nondiabetic children admitted to hospitals in low-income countries (LICs), with a case fatality rate of 16–66%.^3^^–^^8^ The definition of hypoglycemia has been debated as several studies have reported increased mortality risk in children with moderately low blood glucose levels (i.e., “low glycemia” is defined as blood glucose concentrations of 2.5–5.0 mmol/L [variable definition of 2.2–4.4 mmol/L depending on study]), which has a prevalence of 10–28% among sick children in LICs and a case fatality rate of 6–46%.^3^^,^^6^^,^^8^^–^^10^

The SugarFACT trial from Malawi found no effect of the WHO’s recommended treatment of hypoglycemia (5 mL/kg of 10% intravenous [IV] dextrose)^1^ on in-hospital mortality among severely ill children with low glycemia.^11^ This finding raised important questions about potential alternative mechanisms contributing to the excess mortality observed in this group and ways to optimize treatment.

First, there may be an association between low blood glucose concentrations and metabolic acidosis as observed in critically ill children in high-income settings^12^^,^^13^ as well as in those with falciparum malaria in tropical regions.^14^^,^^15^ The role of ketones is of particular interest because of their inverse relationship with glucose levels.^16^ Second, low blood glucose concentrations may be associated with electrolyte derangements because several studies have shown high rates of hypokalemia during hypoglycemic episodes in diabetic patients, potentially leading to fatal cardiac arrhythmias.^17^^,^^18^ Third, there may be more complex interactions linking low glucose levels, electrolyte derangements, metabolic acidosis, and hyperketonemia.

There is a lack of understanding of the complex interplay of various metabolic and electrolyte disturbances among critically ill children in low-income settings. This study aims to assess possible direct or indirect pathways of association between low blood glucose concentrations and electrolyte derangements, metabolic acidosis, and hyperketonemia, hypothesizing that there would be an association between low blood glucose concentrations and electrolytes, metabolic acidosis, and hyperketonemia in children with critical illness.

## MATERIALS AND METHODS

We conducted a cross-sectional study from November 2022 to June 2024 at Queen Elizabeth Central Hospital (QECH), a large government-run tertiary referral hospital in Blantyre, Malawi. All patients ages 0–18 years old arriving at QECH are triaged upon arrival to the Pediatric Accident and Emergency Department (A&E) according to the WHO Emergency Triage Assessment and Treatment (ETAT) guidelines.^19^

Children ages 1–59 months old who were treated in the resuscitation room were screened for inclusion using the following criteria: one or more emergency signs (defined by the WHO as obstructed/absent breathing, central cyanosis, severe respiratory distress, shock/impaired perfusion, coma/reduced consciousness, convulsions ongoing or within 1 hour before arrival to the hospital, or severe dehydration)^19^ and a parent/guardian willing and able to give consent. Exclusion criteria were a known diagnosis of diabetes, known metabolic illness (e.g., adrenal insufficiency or insulinoma), need of trauma care, severe acute malnutrition as per the WHO definition,^1^ blood glucose >10 mmol/L, and previous enrollment in the study. Children with severe acute malnutrition were excluded as they commonly have pronounced glucose and electrolyte derangements compared with well-nourished children because of distinctive underlying physiological mechanisms.^1^ Glucose >10 mmol/L was assumed to be a physiological response to critical illness, which may have inaccurately exaggerated study results.

Recruitment and consenting of study participants were done by a research nurse stationed in the resuscitation room during office hours. Patients with any emergency sign(s) had a blood glucose level checked using a HemoCue Glucose 201 point-of-care device (HemoCue, Ängelholm, Sweden), which has accuracy comparable with laboratory testing.^20^^,^^21^ Children were categorized as hypoglycemic (blood glucose <2.5 mmol/L), low glycemic (2.5–4.9 mmol/L), and normoglycemic (5.0–10.0 mmol/L). Recruitment was done using a 1:4 ratio between hypoglycemic/low glycemic and normoglycemic patients to ensure balanced inclusion of the different groups over time.

Participants had a capillary or venous blood sample taken by the study nurse for a blood gas analysis using an Abbott ISTAT blood gas analyzer with EG6 cartridges (Abbott, North Chicago, IL) and a blood ketone measurement using an Abbott FreeStyle Precision ketone meter. The accuracy of both methods has been previously reported.^22^^,^^23^ These samples were taken before any emergency care was initiated. If this was not possible, the patient was excluded. Study participation did not delay or affect the resuscitation and emergency care received by the included patients. Quality control checks were performed on all devices according to the manufacturer’s instructions every 2 weeks.

Background information about participants was obtained by the study nurse from the parent or guardian in the resuscitation room and when available, from the child’s health passport. WHO emergency signs,^19^ WHO Integrated Management of Childhood Illness (IMCI) danger signs,^24^ vital parameters, consciousness level (Blantyre coma scale),^25^ preliminary diagnosis, and blood test results were recorded. This information was documented at the time of recruitment on electronic clinical record forms using RedCap software on a tablet. A quality check of all collected data was performed at the end of each working day by the responsible study clinician before the data was uploaded to a server.

The primary exposure was glucose level defined as hypoglycemia, low glycemia, or normoglycemia. The outcome variables were plasma levels of potassium, sodium, pH, bicarbonate (HCO_3_), base excess (BE), and ketones in whole blood on arrival at the hospital. The categories for each outcome were defined using a combination of the reference ranges in the i-STAT EG6+ cartridge manual^26^ (Abbott) and the authors’ clinical expertise (Table 1).

**Table 1:** Definitions of glucose, potassium, sodium, ketone, and acidosis (pH, bicarbonate, base excess, and pCO_2_) levels

Variable	Very Low	Low	Normal	High	Very High
Glucose (mmol/L)	<2.5	2.5–4.9	5.0–10.0	–	–
Potassium (mmol/L)	–	<3.5	3.5–4.9	>4.9	–
Sodium (mmol/L)	–	<138	138–146	>146	–
Ketones (mmol/L)	–		<0.6	0.6–2.9	≥3.0

	No/Mild Acidosis	Moderate Acidosis	Severe Acidosis	Metabolic Acidosis	Respiratory Acidosis	Mixed Acidosis
pH	>7.3	7.20–7.30	<7.2	<23.0	–	–
Bicarbonate (mmol/L)	≥18.0	12.0–17.9	<12.0	–	–	<23.0
Base excess (mmol/L)	≥−5	−10 to −6	<−10	–	–	>51
pCO_2_ (mm of Hg)	–	–	–	–	>51	–

pCO_2_ = partial pressure of carbon dioxide.

Baseline characteristics were compared across groups using Kruskal–Wallis tests of median and interquartile range (IQR) for continuous variables and Pearson χ^2^ tests of proportions for categorical variables with a significance level of 0.05. To analyze the association between outcome variables, a multinomial logistic regression analysis model was created. A two-step approach was used. First, we analyzed the adjusted odds of potassium or sodium derangements across categories of glucose, acidosis, and ketone levels and second, analyzed the risk of acidosis across categories of glucose and ketone levels, adjusting for WHO weight-for-age *z*-score and using a significance level of 0.05 in both steps. The required sample size was calculated based on the assumption that the proportions with electrolyte derangement in the hypoglycemic/low glycemic and normoglycemic groups were 20% and 10%, respectively, with a two-sided alpha of 0.05 and 80% power. This resulted in sample sizes of 104 for the hypoglycemic/low glycemic group and 415 for the normoglycemic group.

A model that explored the potential effect of malaria on electrolytes and acidosis was generated by adjusting for malaria (smear or malaria rapid diagnostic test positive) in the multinomial regression model. A sensitivity analysis was conducted that removed patients with respiratory acidosis only. The amount of missing data was <1% (no weight recorded for two patients). Missing values were imputed using multiple imputation and age as a predictor. For subsequent analyses, the first imputed dataset was used as a completed dataset. Data were processed and analyzed using Stata 18 software (StataCorp., College Station, TX).

## RESULTS

**Figure 1.**
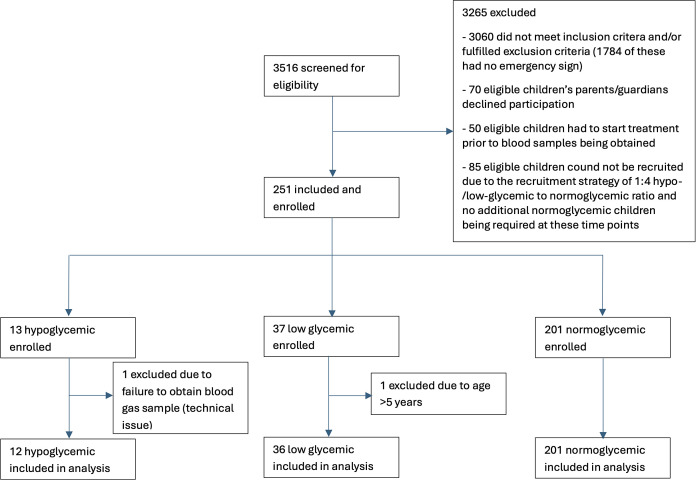
Patient flow.

A total of 3,516 patients were screened, and 251 were recruited, 2 of whom were excluded during analysis (Figure 1). Of those included in the analysis, 12 (4.8%) were hypoglycemic, 36 (14.5%) were low glycemic, and 201 (80.7%) were normoglycemic. The median (IQR) age was 25.9 (13.0–37.0) months old, and the median weight was 10.0 (8.2–12.9) kg. A total of 148 males (59%) and 101 females (41%) were included; 197 patients (79%) had been referred from other health care facilities, and 196 patients (79%) had received treatment before arrival to hospital, most commonly antimalarial treatment (49%) and antibiotics (47%). Fifteen patients (6.0%) received IV fluids and seven patients (2.8%) received oral rehydration solution before arrival. Twenty-three patients (9%) had a known chronic condition.

The most prevalent emergency sign was convulsions (57%) followed by respiratory distress (34%) and reduced consciousness (26%). Seventy-two patients (29%) had multiple emergency signs. The most common preliminary diagnoses were malaria (48%), central nervous system infection (26%), pneumonia (18%), sepsis (13%), and gastroenteritis (12%). Emergency signs were similarly distributed between the glucose-level groups apart from reduced consciousness being more common in the hypoglycemic and low glycemic patients compared with the normoglycemic ones (*P* <0.001). A positive malaria test was more common among the low glycemic patients (*P* = 0.035) as well as signs of shock (*P* = 0.031) and clinical signs of anemia (*P* = 0.007). The preliminary diagnoses were relatively evenly distributed between the glucose categories apart from sepsis being more common in the hypoglycemic/low glycemic groups (*P* = 0.031) and infectious gastroenteritis being more common in the hypoglycemic group only (*P* = 0.034). The children with hypoglycemia and low glycemia were more likely to have IMCI danger signs (*P* = 0.001) and a longer duration since last feed (*P* <0.001) (Table 2).

**Table 2:** Background characteristics across glucose categories

Variable	Normoglycemic	Low Glycemic	Hypoglycemic	Total	*P*-Value
*N*	201 (80.7%)	36 (14.5%)	12 (4.8%)	249 (100.0%)	–
Age (months)	23.0 (10.0–36.0)	36.0 (23.5–45.5)	23.0 (18.5–32.5)	25.0 (13.0–37.0)	0.003
Sex					
Male	118 (58.7%)	26 (72.2%)	4 (33.3%)	148 (59.4%)	0.053
Female	83 (41.3%)	10 (27.8%)	8 (66.7%)	101 (40.6%)	
Weight (kg)	10.0 (7.9–12.5)	11.2 (9.2–13.8)	10.4 (10.0–12.0)	10.0 (8.2–12.9)	0.089
Referral	156 (77.6%)	30 (83.3%)	11 (91.7%)	197 (79.1%)	0.405
Received prior treatment of current illness	157 (78.1%)	28 (77.8%)	11 (91.7%)	196 (78.7%)	0.531
Antibiotic (oral/intravenous/intramuscular)	95 (47.3%)	14 (38.9%)	8 (66.7%)	117 (47.0%)	0.244
Antimalaria (oral/intravenous/intramuscular)	93 (46.3%)	21 (58.3%)	9 (75.0%)	123 (49.4%)	0.079
Oral rehydration solution	6 (3.0%)	1 (2.8%)	0 (0.0%)	7 (2.8%)	0.831
Intravenous fluids	13 (6.5%)	1 (2.8%)	1 (8.3%)	15 (6.0%)	0.653
Other*	97 (48.3%)	19 (52.8%)	5 (41.7%)	121 (48.6%)	0.782
Duration since last feed (hours)	5.0 (1.0–12.0)	13.0 (4.0–18.0)	16.0 (15.0–18.0)	5.0 (2.0–15.0)	<0.001
Duration of illness (days)	2.0 (2.0–4.0)	3.0 (2.0–4.0)	2.0 (1.0–5.0)	3.0 (2.0–4.0)	0.552
Any chronic conditions^†^	17 (8.5%)	6 (16.7%)	0 (0.0%)	23 (9.3%)	0.157
Emergency sign(s)					
Obstructed/absent breathing	2 (1.0%)	0 (0.0%)	0 (0.0%)	2 (0.8%)	0.786
Respiratory distress	69 (34.3%)	13 (36.1%)	2 (16.7%)	84 (33.7%)	0.430
Central cyanosis	2 (1.0%)	0 (0.0%)	0 (0.0%)	2 (0.8%)	0.786
Shock/impaired perfusion	2 (1.0%)	1 (2.8%)	0 (0.0%)	3 (1.2%)	0.616
Reduced consciousness	43 (21.4%)	19 (52.8%)	3 (25.0%)	65 (26.1%)	<0.001
Convulsions	118 (58.7%)	19 (52.8%)	5 (41.7%)	142 (57.0%)	0.438
Severe dehydration	18 (9.0%)	3 (8.3%)	3 (25.0%)	24 (9.6%)	0.180
IMCI danger sign(s) present	77 (38.3%)	25 (69.4%)	7 (58.3%)	109 (43.8%)	0.001
Hypoxemia (SpO_2_ < 90%)	8 (4.0%)	1 (2.8%)	0 (0.0%)	9 (3.6%)	0.741
Clinical signs of anemia	33 (16.4%)	14 (38.9%)	2 (16.7%)	49 (19.7%)	0.007
Malaria positive (smear or MRDT)	63 (31.3%)	19 (52.8%)	3 (25.0%)	85 (34.1%)	0.035
Preliminary diagnosis					
Malaria	94 (46.8%)	21 (58.3%)	5 (41.7%)	120 (48.2%)	0.396
Lower respiratory tract infections	60 (29.9%)	8 (22.2%)	0 (0.0%)	46 (18.5%)	0.214
Infectious gastroenteritis	23 (11.4%)	2 (5.6%)	4 (33.3%)	29 (11.6%)	0.034
Sepsis	21 (10.4%)	7 (19.4%)	4 (33.3%)	32 (12.9%)	0.031
Meningitis	55 (27.4%)	6 (16.7%)	4 (33.3%)	65 (26.1%)	0.341
Bronchiolitis	22 (10.9%)	0 (0.0%)	0 (0.0%)	22 (8.8%)	0.056
Epilepsy	12 (6.0%)	2 (5.6%)	0 (0.0%)	14 (5.6%)	0.684
Febrile seizure	14 (7.0%)	4 (11.1%)	0 (0.0%)	18 (7.2%)	0.414
Other^‡^	31.1 (24.7–37.2)	14 (38.9%)	4 (33.3%)	92 (36.9%)	0.938

IMCI = Integrated Management of Childhood Illness; MRDT = malaria rapid diagnostic test; SpO_2_ = peripheral oxygen saturation. Median, interquartile range, and Kruskal–Wallis test are reported for continuous variables. Frequency, percentage, and Pearson’s χ^2^ test are reported for categorical variables.

* In descending order, other includes antipyretics, anticonvulsants, salbutamol nebulizers, diuretics, potassium oral, zinc, adrenaline, and morphine.

^†^ In descending order, chronic conditions include cerebral palsy, epilepsy, HIV, congenital heart disease, asthma, developmental delay, hydrocephalus, cardiomyopathy, and viral-induced wheeze.

^‡^ In descending order, other includes viral-induced wheeze, acute kidney injury, congenital heart disease, cardiac failure/pulmonary edema, tuberculosis, space-occupying lesion, and vocal cord papilloma.

In this study sample, a total of 182 patients (73.1%) had an electrolyte disturbance. Hyperkalemia (26.9%) was the most prevalent electrolyte disturbance closely followed by hyponatremia (21.3%), hypernatremia (10.0%), and hypokalemia (8.0%). There was no significant difference in the levels of potassium and sodium between the glucose-level groups (*P* = 0.233 and *P* = 0.654, respectively). A total of 215 patients (86.3%) had acidosis; of these patients, 206 (95.8%) had metabolic acidosis only, 8 (3.9%) had mixed metabolic and respiratory acidosis, and 5 (2.4%) had respiratory acidosis only. The patients in the hypoglycemic and low glycemic groups had significantly lower pH, HCO_3_, and BE as well as significantly higher ketones compared with those with normoglycemia (*P* <0.001). Although all groups to some extent appeared to compensate for metabolic acidosis with low partial pressure of carbon dioxide (pCO_2_), the low glycemic group had significantly lower pCO_2_ compared with the other two groups (*P* <0.001) (Table 3). In 91 patients (36.5%), blood gases were capillary. In 91 patients (36.5%) blood gases were capillary, in 115 patients (46.2%) venous and in 43 patients (17.3%) of unknown type. The blood gas types were evenly distributed across glucose categories (*P* = 0.539).

**Table 3:** pH, pCO_2_, bicarbonate, base excess, sodium, potassium, ketones, and hematocrit across glucose categories

Variable	Normoglycemic	Low Glycemic	Hypoglycemic	Total	*P*-Value
*N*	201 (80.7%)	36 (14.5%)	12 (4.8%)	249 (100.0%)	–
pH	7.4 (7.3–7.4)	7.3 (7.2–7.4)	7.3 (7.2–7.4)	7.4 (7.3–7.4)	<0.001
pCO_2_ (mm of Hg)	31.1 (24.7–37.2)	23.6 (17.1–29.4)	31.3 (26.6–38.6)	29.9 (23.9–36.7)	<0.001
Bicarbonate (mmol/L)	19.8 (15.8–22.3)	12.1 (7.6–14.8)	15.3 (11.4–19.6)	18.8 (13.4–21.8)	<0.001
Base excess (mmol/L)	−5.0 (−9.0 to −2.0)	−14.0 (−20.0 to −10.0)	−10.5 (−15.5 to −5.5)	−6.0 (−12.0 to −3.0)	<0.001
Potassium (mmol/L)	4.5 (4.1–5.0)	4.8 (4.1–5.9)	4.5 (3.7–5.5)	4.6 (4.1–5.1)	0.233
Sodium (mmol/L)	139.0 (135.0–141.0)	139.0 (134.0–141.0)	139.0 (137.5–141.0)	139.0 (135.0–141.0)	0.654
Ketones (mmol/L)	0.9 (0.2–2.0)	2.7 (1.1–4.8)	3.8 (2.4–4.5)	1.1 (0.4–2.4)	<0.001
Hematocrit (%)	34.0 (31.0–37.0)	28.0 (17.0–35.0)	32.5 (26.0–36.0)	34.0 (29.0–37.0)	0.002

Median, interquartile range, and Kruskal–Wallis test are reported for continuous variables. pCO_2_ = partial pressure of carbon dioxide.

In the model with potassium as the outcome, there were increased odds of hypokalemia in children with severe acidosis (adjusted odds ratio [aOR] = 24.5, 95% CI: 2.91–205.88) compared with those with no/mild acidosis adjusted for glucose categories and ketone level as well as weight-for-age *z*-score. For those with moderate acidosis, there was a tendency toward increased odds of hypokalemia that did not pass significance level, but there was no increased risk of hyperkalemia in either group. The model with sodium as the outcome showed an association of acidosis with increased odds of hyponatremia (aOR = 2.58, 95% CI: 1.33–4.99 for moderate acidosis and aOR = 3.62, 95% CI: 1.71–7.68 for severe acidosis) and hypernatremia (aOR = 9.42, 95% CI: 2.50–35.5 for severe acidosis). There were no increased odds of potassium or sodium derangements in hypoglycemic and low glycemic children compared with normoglycemic ones. However, there was increased odds of acidosis in the low glycemic group (aOR = 5.84, 95% CI: 1.17–29.2 for moderate acidosis and aOR = 27.1, 95% CI: 6.08–120.9 for severe acidosis), which was not the case for the hypoglycemic group. Children with very high ketones had higher odds of severe acidosis (aOR = 3.02, 95% CI: 1.20–7.60) (Table 4). Adjusting for malaria positivity provided similar results to the primary analysis, although a diagnosis of malaria appeared to be associated with reduced odds of hyperkalemia (aOR = 0.46, 95% CI: 0.25–0.85). Finally, the sensitivity analysis done by removing the five patients with respiratory acidosis provided no significant changes to the results from the initial model (Supplemental Appendix 1).

**Table 4:** Multinomial regression analysis for association between blood glucose levels/acidosis levels/ketone levels/weight-for-age *z*-score and deranged potassium (A) and sodium (B) respectively, as well as association between blood glucose level/ketone level and moderate/severe acidosis (C) (*N* = 249).

A					B					C				
Variable	aOR	*P*-Value	95% CI		Variable	aOR	*P*-Value	95% CI		Variable	aOR	*P*-Value	95% CI	
Normal potassium	(Base outcome)				Normal sodium	(Base outcome)				No/mild acidosis	(Base outcome)			
Hypokalemia					Hyponatremia					Moderate acidosis				
Normoglycemia	1.00					1.00					1.00			
Low glycemia	0.50	0.356	0.11	2.20		0.78	0.566	0.33	1.84		5.84	0.032	1.17	29.22
Hypoglycemia	0.49	0.533	0.05	4.71		0.28	0.080	0.07	1.16		2.91	0.237	0.49	17.18
No/mild acidosis	1.00					1.00								
Moderate acidosis	7.77	0.064	0.89	68.02		2.58	0.005	1.33	4.99					
Severe acidosis	24.48	0.003	2.91	205.88		3.62	0.001	1.71	7.68					
Normal ketones	1.00					1.00					1.00			
High ketones	3.99	0.091	0.80	19.85		1.14	0.701	0.59	2.20		1.39	0.367	0.68	2.86
Very high ketones	1.90	0.473	0.33	10.87		0.87	0.718	0.42	1.82		1.34	0.492	0.58	3.11
Weight-for-age *z*-score	0.74	0.091	0.52	1.05		1.10	0.305	0.92	1.32		1.06	0.568	0.86	1.30
Hyperkalemia					Hypernatremia					Severe acidosis				
Normoglycemia	1.00					1.00					1.00			
Low glycemia	1.59	0.286	0.68	3.74		0.65	0.547	0.17	2.60		27.11	0.000	6.08	120.89
Hypoglycemia	0.60	0.485	0.14	2.51		5.62e-07	0.985	–	–*		4.69	0.073	0.87	25.30
No/mild acidosis	1.00					1.00								
Moderate acidosis	0.58	0.111	0.30	1.13		2.24	0.283	0.51	9.73					
Severe acidosis	0.95	0.901	0.46	1.97		9.42	0.001	2.50	35.51					
Normal ketones	1.00					1.00					1.00			
High ketones	1.16	0.655	0.61	2.22		4.98	0.046	1.03	24.13		2.29	0.056	0.98	5.36
Very high ketones	0.98	0.965	0.47	2.03		1.78	0.521	0.31	10.39		3.02	0.019	1.20	7.60
Weight-for-age *z*-score	0.85	0.072	0.71	1.01		1.03	0.864	0.73	1.44		0.95	0.662	0.76	1.19

A = association between blood glucose levels/acidosis levels/ketone levels/weight-for-age *z*-score and deranged potassium; aOR = adjusted odds ratio; B = association between blood glucose levels/acidosis levels/ketone levels/weight-for-age *z*-score and deranged sodium; C = association between blood glucose level/ketone level and moderate/severe acidosis.

* There were insufficient (*n* = 0) patients in this category to calculate CI.

## DISCUSSION

This exploratory cross-sectional study observed an association between low glucose levels and moderate to severe metabolic acidosis in critically ill children in Malawi as well as an association between metabolic acidosis and hypokalemia, hyponatremia, and hypernatremia. Although hyperketonemia did not appear to play a central role in these pathways when adjusted for in the regression model, it was independently associated with both low blood glucose concentrations and metabolic acidosis. Contrary to the initial hypothesis, no direct association was found between low blood glucose concentrations and potassium or sodium derangements.

An association was found between low glycemia and moderate to severe metabolic acidosis. Although this association was not observed in the hypoglycemic group, possibly because of the small sample size in that category (*n* = 12), its presence in the larger low glycemica group is of clinical relevance as it supports the notion that the current WHO cutoff for hypoglycemia may be too low for mortality risk prediction. Furthermore, the low glycemic group appeared to have better respiratory compensation of metabolic acidosis compared with the hypoglycemic group. These findings align with studies in children with malaria, which have shown associations between hypoglycemia, metabolic acidosis, and hyperlactatemia—each of these correlating with increased morbidity and mortality.^14^^,^^15^^,^^27^ Although this may simply reflect the severity of underlying illness, studies suggest that the morbidity and mortality associated with metabolic acidosis are more closely related to the predominant anion and underlying pathology than to the acidosis itself.^28^^,^^29^

In this study, hyperketonemia was more common among children with low blood glucose concentrations compared with those with normal glucose levels, likely reflecting prolonged fasting and underlying macronutrient deficiency. Elevated ketone levels were associated with an increased risk of severe acidosis, which was expected given the acidic nature of ketone bodies. Previously, it was unclear whether critically ill children could mount a ketogenic response to illness-induced macronutrient deficiency. However, our findings are consistent with data from high-income settings, suggesting that ketogenesis does occur in critically ill pediatric patients,^30^ despite presumably poorer underlying nutritional status in our study population. Other anions, such as lactate, were not measured in this study, and future research should aim to assess the contribution of these additional anions to the metabolic disturbances observed in this patient population.

Electrolyte derangements are common in critically ill children^31^^,^^32^ and therefore, could be expected to coincide with low blood glucose concentrations. However, our findings did not show a direct association between low glucose levels and potassium or sodium abnormalities. The hypokalemia typically observed in diabetic patients during hypoglycemic episodes^17^^,^^18^ was not evident in children with hypoglycemia or low glycemia in our study population, possibly because of other underlying etiologies. In addition, a proportion of patients may have had pre-existing conditions that increased their susceptibility to specific electrolyte disturbances. Previous studies have shown associations between malaria and renal failure with hypokalemia and hyperkalemia as well as malaria and sepsis with hyponatremia in pediatric patients.^33^^–^^36^

Over one third of patients in this study were malaria positive. Pediatric severe malaria is commonly accompanied by acidosis and electrolyte derangements, which may be further complicated by acute kidney injury (AKI).^36^^,^^37^ Although renal function was not specifically assessed in this study, adjusting for malaria positivity found an association between malaria and reduced risk of hyperkalemia but no significant impact on hypokalemia or sodium levels. Thus, a malaria diagnosis and possible secondary AKI did not substantially influence the overall results.

Our results revealed a notable pattern of potassium derangement across all levels of acidosis: an increased risk of hypokalemia in both moderate and severe acidosis and a reduced risk of hyperkalemia in moderate acidosis, although this was not observed in severe acidosis. Several factors may explain why the typical association between metabolic acidosis and hyperkalemia was not evident in our study population. The type of contributing anion influences potassium levels, and lactic acidosis is commonly associated with hypokalemia.^38^ Although lactate was not measured in our sample, it is reasonable to presume that a substantial proportion of patients had lactic acidosis because of tissue hypoperfusion related to critical illness and the underlying presumed etiologies. However, without lactate measurements, it remains uncertain which anion is primarily responsible for the metabolic acidosis and subsequent potassium shifts in our study sample and thus, prevents us from making definite conclusions regarding the underlying mechanism. In addition, total body potassium depletion because of gastrointestinal or urinary losses along with factors such as the effects of insulin, catecholamines, and hypertonicity may also have influenced potassium distribution.^29^^,^^38^

Sodium homeostasis appeared to be significantly associated with the degree of metabolic acidosis in this study. There were progressively increased odds of hyponatremia in moderate and severe acidosis as well as increased odds of hypernatremia in severe acidosis. However, it is not possible to draw any conclusions about a direct relationship between these factors as they are likely to have been affected by a multitude of pathways involved in acid–base and sodium balance. Although sodium homeostasis is primarily regulated by antidiuretic hormone, atrial natriuretic peptide, and aldosterone, metabolic acidosis may contribute to hyponatremia by reducing sodium reabsorption in the proximal tubule of the kidney. Hypernatremia, by contrast, is likely to have resulted only from other unrelated factors, such as dehydration, fluid shifts, AKI, and prior treatments received.^29^ Finally, although our results contradict the initial hypothesis of an association between low glucose concentrations and electrolyte disturbance, they should be interpreted with caution as they may simply reflect the underpowering of the hypoglycemic subgroup rather than definitive evidence of no effect.

The main limitation of this study was failure to reach the target sample size. More specifically, we had a small sample of hypoglycemic children resulting from slower than anticipated recruitment and the practical difficulties of obtaining blood samples before administering treatment in severely sick children. We may, therefore, have been underpowered to detect and reject significant associations. In addition, the unequal size of the groups created challenges for statistical interpretation. Although the results indicate statistically significant associations with some strikingly low *P*-values considering the small sample, the breadth of CIs for some parameters highlights a lack of precision, and therefore, the magnitude of effect for these should be interpreted with caution.

Point-of-care devices were used to measure glucose and electrolyte levels, utilizing a mix of capillary and venous blood samples. This may have introduced minor inaccuracies, particularly for values near the classification cutoffs, potentially leading to misclassification in glucose, electrolytes, and blood gas categories. Potassium levels, in particular, may have been falsely elevated because of sample hemolysis.^33^ These devices were used for practical reasons and reflect the analysis methods that could be available in LICs. Furthermore, the lack of renal function and lactate measurements prevented definitive conclusions from being made about the underlying mechanisms behind the findings.

The study’s main strength was the heterogenicity of the sample, which reflects the clinical reality in Malawi and is representative of the underlying source population, allowing the results to be generalized to this setting. However, this diversity also introduces potential confounding factors and effect modifiers, which were difficult to adjust for because of the wide variety of underlying conditions present in the study sample. Future research with larger sample sizes should aim to confirm the relationships between low blood glucose, a broader range of electrolytes and anions, and their impact on morbidity and mortality.

## CONCLUSION

In conclusion, we found an association between low glucose levels and metabolic acidosis as well as an association between metabolic acidosis and hypokalemia, hyponatremia, and hypernatremia in our study sample of critically ill children in a low-income setting. This exploratory study contributes to the existing body of research by highlighting the complex interplay of metabolic disturbances in critically ill pediatric patients in LICs, and additionally, it identifies potential avenues for future investigation.

## Supplemental Materials

10.4269/ajtmh.25-0635 Supplemental Materials
