# Supplementary material for: Low Blood Glucose and Its Relation to Electrolyte Derangements and Metabolic Acidosis in Critically Ill Children in Malawi
Source: Am J Trop Med Hyg. 2026 Mar 31;114(5):981–8. doi: 10.4269/ajtmh.25-0635 (PMC13153582; doi:10.4269/ajtmh.25-0635)
Supplement: Supplemental Materials [file tpmd250635.SD1.pdf]

Appendix 1. Multinomial regression analysis with sensitivity analysis, excluding patients with respiratory acidosis, for association between blood glucose levels/acidosis levels/ketone levels/weight-for-age z-score and deranged potassium and sodium respectively, as well as association between blood glucose level/ketone level and moderate/severe acidosis (n=249).

	aOR	p-value	[95% conf. interval]			aOR	p-value	[95% conf. interval]			aOR	p-value	[95% conf. interval]	
Normal potassium	(base outcome)				*Normal sodium*	(base outcome)				*No/mild acidosis*	(base outcome)			
Hypokalemia					*Hyponatremia*					*Moderate acidosis*				
Normoglycemia	1.00					1.00					1.00			
Low glycemia	0.49	0.356	0.11	2.21		0.79	0.579	0.33	1.85		16.95	0.007	2.18	131.54
Hypoglycemia	0.52	0.579	0.05	5.19		0.26	0.064	0.06	1.08		6.01	0.102	0.70	51.57
No/mild acidosis	1.00					1.00								
Moderate acidosis	6.42	0.088	0.76	54.24		3.89	0.000	2.02	7.50					
Severe acidosis	31.94	0.002	3.57	285.99		4.94	0.000	2.06	11.88					
Normal ketones	1.00					1.00					1.00			
High ketones	3.11	0.172	0.61	15.80		1.06	0.864	0.53	2.12		1.42	0.316	0.71	2.84
Very high ketones	1.46	0.677	0.25	8.60		0.75	0.460	0.35	1.61		1.76	0.163	0.80	3.90
Weight-for-age z-score	0.70	0.064	0.47	1.02		1.06	0.535	0.88	1.28		1.05	0.612	0.86	1.29
Hyperkalemia					*Hypernatremia*					*Severe acidosis*				
Normoglycemia	1.00					1.00					1.00			
Low glycemia	1.59	0.288	0.67	3.76		0.78	0.730	0.20	3.13		48.05	0.000	6.00	384.76
Hypoglycemia	0.63	0.533	0.15	2.68		1.22e-06	0.979	.	.*		7.76	0.077	0.80	75.19
No/mild acidosis	1.00					1.00								
Moderate acidosis	0.48	0.025	0.25	0.91		3.88	0.066	0.92	16.47					
Severe acidosis	1.14	0.761	0.49	2.66		12.08	0.001	2.62	55.71					
Normal ketones	1.00					1.00					1.00			
High ketones	1.22	0.571	0.62	2.40		8.35	0.049	1.01	68.95		4.66	0.008	1.51	14.42
Very high ketones	1.13	0.745	0.52	2.40		2.87	0.361	0.30	27.59		5.48	0.006	1.64	18.34
Weight-for-age z-score	0.87	0.130	0.72	1.04		0.99	0.947	0.69	1.42		0.98	0.872	0.75	1.27

*Insufficient (n=0) patients in this category to calculate CI.
